# Cohort Profile: South African Population Research Infrastructure Network (SAPRIN)

**DOI:** 10.1093/ije/dyab261

**Published:** 2021-12-30

**Authors:** Mark A Collinson, Taurayi Mudzana, Tinofa Mutevedzi, Kathleen Kahn, Eric Maimela, F Xavier Gómez-Olivé, Thobeka Mngomezulu, Dickman Gareta, Chodziwadziwa W Kabudula, Rathani Nemuramba, Joseph Tlouyamma, Stephen Tollman, Kobus Herbst

**Affiliations:** Department of Science and Innovation, South African Population Research Infrastructure Network (SAPRIN), South African Medical Research Council, South Africa; MRC/Wits Rural Public Health and Health Transitions Research Unit, School of Public Health, University of the Witwatersrand, Johannesburg, South Africa; Department of Science and Innovation, South African Population Research Infrastructure Network (SAPRIN), South African Medical Research Council, South Africa; Department of Science and Innovation, South African Population Research Infrastructure Network (SAPRIN), South African Medical Research Council, South Africa; MRC/Wits Rural Public Health and Health Transitions Research Unit, School of Public Health, University of the Witwatersrand, Johannesburg, South Africa; DIMAMO Population Health Research Centre, University of Limpopo, Turfloop, South Africa; MRC/Wits Rural Public Health and Health Transitions Research Unit, School of Public Health, University of the Witwatersrand, Johannesburg, South Africa; Africa Health Research Institute, Mtubatuba, South Africa; Africa Health Research Institute, Mtubatuba, South Africa; MRC/Wits Rural Public Health and Health Transitions Research Unit, School of Public Health, University of the Witwatersrand, Johannesburg, South Africa; DIMAMO Population Health Research Centre, University of Limpopo, Turfloop, South Africa; DIMAMO Population Health Research Centre, University of Limpopo, Turfloop, South Africa; MRC/Wits Rural Public Health and Health Transitions Research Unit, School of Public Health, University of the Witwatersrand, Johannesburg, South Africa; Department of Science and Innovation, South African Population Research Infrastructure Network (SAPRIN), South African Medical Research Council, South Africa; Africa Health Research Institute, Mtubatuba, South Africa

Key Features

- The South African Population Research Infrastructure Network (SAPRIN) is a harmonized national network of health and demographic surveillance system (HDSS) nodes, constituted in 2016 with the aim of establishing a national resource and research platform to strengthen the evidence for informing health and social policy and programming at a time of stringent fiscal constraint.
- The three HDSS founding nodes are hosted by the Medical Research Council/Wits Rural Public Health and Health Transitions Research Unit (Agincourt) in Mpumalanga province, the University of Limpopo (DIMAMO) in Limpopo province and the Africa Health Research Institute (AHRI) in KwaZulu-Natal.
- In 2020, the SAPRIN population cohort covered 355 000 individuals for whom data on births and deaths, residency status and migration, household asset ownership status, and measures of well-being including employment, education and social protection are updated three times a year.
- Attrition is low due to keeping track of population dynamics, and refusal rates are usually below 1%.
- The SAPRIN longitudinal population dataset is publicly available at [http://saprindata.samrc.ac.za].

## Why was the cohort set up?

South Africa is striving to emerge from a legacy of gross social injustice and consequent health and socioeconomic inequality, to becoming a country where all residents have opportunities to build productive lives. However, recent declines in economic performance and unemployment, exacerbated by weaknesses in national and provincial level governance, coupled with colliding epidemics of HIV/AIDS and non-communicable diseases, have left the country’s leadership with serious, seemingly intractable challenges. Moreover as with most countries, the effects of stringent sociobehavioural responses to the coronavirus disease 2019 (COVID-19), with serious economic consequences, serve to amplify such challenges.

At a national level, there has been a realization that reliable, relevant, and long-term research infrastructure is needed to generate data and evidence to underpin policies that can address these interconnected problems. The Department of Science and Innovation invested in a programme of national research infrastructure development, called the South African Research Infrastructure Roadmap.^1^ This includes the South African Population Research Infrastructure Network (SAPRIN) established in 2016. Building on the strengths of three existing Health and Demographic Surveillance Systems, SAPRIN is a harmonized network of population-based cohorts with data that are made accessible for the research community and national policy makers. The aim of the network is to provide: (i) ongoing, up-to-date, longitudinal health, demographic and socioeconomic data, representative of South Africa’s fast-changing poorer communities, for research and policy evaluation and interpretation and calibration of national data; (ii) an extensive, versatile and interdisciplinary research platform for researchers from universities, science councils and regional and international collaborations; (iii) a scientific evidence base for cost evaluation, policy making and targeting intervention programmes; and (iv) expanded human capacity for conducting research that is effectively linked with national, regional and international networks.

## What has been done?

Since SAPRIN’s inception in 2016, three existing health and demographic surveillance system (HDSS) nodes in South Africa have been brought together to form a harmonized national network, namely: MRC/Wits University Agincourt HDSS in Bushbuckridge District, Mpumalanga, established in 1992 with a 2019 population of 115 000 individuals^2^; University of Limpopo DIMAMO HDSS in the Capricorn District of Limpopo, established in 1995, with population of 8000 individuals, then expanded to 42 000 individuals in 2010, and expanded again in 2019 to 100 000^3^; and the Africa Health Research Institute (AHRI) HDSS in uMkhanyakude District, KwaZulu-Natal, linked to the University of KwaZulu-Natal,^4^ established in 2000 with a 2019 population of 140 000 individuals. The three founding nodes added to their capacities as needed to introduce a common scientific protocol. In 2019, SAPRIN released its first harmonized multicentre dataset, including the legacy data from the start of each of the founding three HDSS nodes.

**Figure 1:**
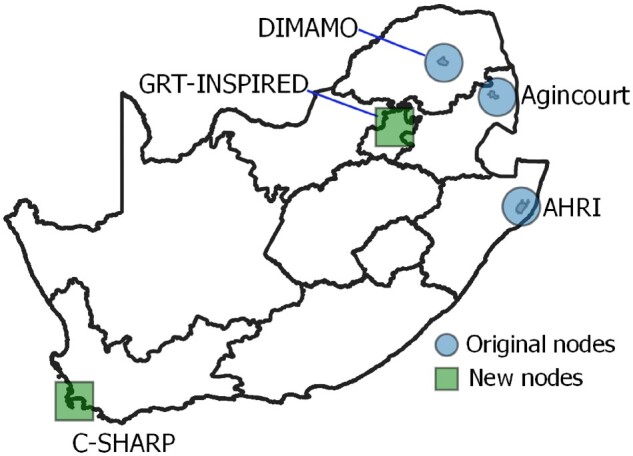
Representational map of SAPRIN, showing both founding and joining nodes in the South African setting

The population platform will be expanded to include urban nodes in the country’s two main metropolitan areas, Gauteng and the Western Cape. The new Gauteng node, GRT-INSPIRED, was established in 2020 and is run by a coalition of three universities, namely University of Witwatersrand, University of Johannesburg and University of Pretoria. With a common scientific protocol in place, the new HDSS nodes will be able to produce data harmonized with the existing platform. The Western Cape node, C-Sharp, will start in 2022. The SAPRIN aim, with long-term support from the Department of Science and Innovation, is to run seven nodes across South Africa which will incorporate dynamic, bi-directional migration flows linking poor rural communities with urban centres (see Figure 1).

## Who is in the cohort?

Each SAPRIN node is a fully functioning Health and Demographic Surveillance System (HDSS). Such ‘whole population cohorts’ provide longitudinal platforms that enable rigorous individual follow-up, systematic recording of all vital events (births, deaths, migrations) and the opportunity for a range of observational and interventional study designs.^2–4^

Individuals eligible for inclusion are all members of the households located within the stipulated geographical boundaries of a SAPRIN node—usually comprising several thousand households in an area conforming to the boundaries for decentralized health and social administration.

### Defining household membership

Households and household membership are self-defined by a household informant in an interview at their place of residence (or a telephone interview). A resident household member is defined as an individual who intends to sleep most of the time at the dwelling place of the household, over a 4-month period. A non-resident member is defined as an individual who retains close economic and relationship ties to the household but does not physically reside with them for the majority of time.

### Registering vital events

To ensure complete household membership, a rigorous system is in place for routinely updating all vital events including in- and out-migration, births and deaths of individuals in the population cohort. In about half of the cases, individuals tend to have a single period of residence from the time of first enumeration, at birth or in-migration, to their eventual death or out-migration. In the remaining cases, household members tend to transition from resident to non-resident through out-migration or movement within the HDSS area. Individuals returning from a period of non-residency continue with a new residency period and retain their original identification. Migration reconciliation takes place to ensure that new migrants are verified against known individuals, to prevent duplicate counting for the same individual.

## How often have they been followed up?

SAPRIN nodes collect data in three annual rounds, over a 45-week schedule: one by computer-assisted personal face-to-face interview and two telephone interviews. Frequency of follow-up is designed to minimize the risk of missing demographic events, especially pregnancies, which allows collection of pregnancy outcomes including stillbirths and neonatal deaths. Before 2019, the three HDSS nodes had different data collection frequencies, influenced by the scientific questions addressed as well as resources available, as shown in Table 1. Attrition is low due to keeping track of population dynamics. Refusal rates are low and usually below 1%.

**Table 1:** South African Population Research Infrastructure Network (SAPRIN) infrastructural development stages

Year	Longitudinal, population research infrastructure	Population in surveillance	Type of informant	Frequency of rounds per year
**Single-centre Health and Demographic Surveillance System (HDSS) nodes**				
1992	Agincourt Health and Demographic Surveillance System starts	70 000 individuals 15 000 households	Household proxy	1
1995	Dikgale HDSS starts	8000 individuals	Household—proxy	1
2000	Africa Centre Demographic Information System starts	85 000 individuals	Household—proxy Individual	3 1
2010	Dikgale HDSS expands	42 000 individuals	Household—proxy	1
	Africa Centre Demographic Information System expands	100 000 individuals	Household—proxy Individual	3 1
	Agincourt HDSS expands	80 000 individuals	Household—proxy	1
2016	Africa Centre Demographic Information System becomes Africa Health Research Institute (AHRI), Population Intervention Programme	125 000 individuals	Household—proxy Individual	3 1
**Multicentre HDSS network**				
2016	SAPRIN initiated as national research infrastructure hosted by the SAMRC	297 000 individuals		
	Agincourt HDSS node	115 000 individuals	Household proxy	1
	Dikgale HDSS node	42 000 individuals	Household proxy	1
	AHRI HDSS node	140 000 individuals	Household proxy Individual	3 1
2018	Dikgale becomes DIMAMO HDSS and expands	100 000 individuals	Household proxy	1
2019	SAPRIN first public data release—harmonized multicentre legacy data	355 000 individuals	Household—proxy	
	Agincourt HDSS node	115 000 individuals	Household—proxy	1
	DIMAMO HDSS node	100 000 individuals	Household—proxy	3
	AHRI HDSS node	140 000 individuals	Household—proxy Individual	3 1
2020	GRT-INSPIRED			
2022	C-SHARP			

Dikgale HDSS, the forerunner of DIMAMO HDSS; Africa Centre DIS, a forerunner of Africa Health Research Institute, Population Intervention Programme; SAMRC, South African Medical Research Council; DIMAMO, an amalgam of the initial two letters of the three sub-districts covered by the field-site; GRT-INSPIRED, the Gauteng Research Triangle Initiative for the Study of Population, Infrastructure and Regional Economic Development; C-SHARP, Cape Town Surveillance through Healthcare Action Research Project.

## What has been measured?

Through ongoing field-, telephone- and computer-based operations, the following individual and household indicators are regularly updated: births and deaths, residence and in- and out-migrations, which are updated three times per year; and measures of well-being, including household asset status, labour status, education and social protection, which are updated once per year. Each node follows the core protocol, summarized in Table 2 with more detail given in the Supplementary material, available as Supplementary data at *IJE* online. This forms the basis for the SAPRIN longitudinal population dataset. The public access databases are structured to enable longitudinal analysis of events occurring in time. Three types of dataset are available and updated each year: a basic residency file; a file with residencies divided by age, year and delivery date; and a file with time-varying attributes, such as education, employment, marital status and household asset status. Household asset measures include 35 ordinal variables on asset ownership in the categories of modern assets, livestock, power supply, house construction materials and water and sanitation. A household socioeconomic status indicator is constructed by normalizing the sum of scores in each category and summing the five category scores.^5^

**Table 2:** Variables^a^ collected at South African Population Research Infrastructure Network (SAPRIN) Health and Demographic Surveillance System nodes

Core variables	Location, type of dwelling, household identifier, individual details
Events	In-migration, out-migration, birth, pregnancy outcome, death, change in household membership, change in head of household, union (such as marriage or separation
Episodes	Duration of residence, duration of household membership, details of informal and formal conjugal relationships, details of pregnancies, government social supports
Status observation	Individual: education, employment, health status, medical conditions, healthcare utilization, vaccination history
	Household: dwelling construction, source of drinking water, sanitation, fuel for cooking, food security, assets (such as telephone, fridge, sewing machine), financial situation, victim of crime (such as theft, assault)
COVID-19 pandemic	Knowledge about pandemic, source of information, household and individual actions, employment, access to health care

a Full list of variables given in the Supplementary material, available as Supplementary data at *IJE* online.

In response to the emergence of the COVID-19 epidemic, in April 2020 SAPRIN implemented an active surveillance module in a random sample of more than 2000 households in the three nodes in Mpumalanga, Limpopo and KwaZulu-Natal. Respondents have been interviewed every 2–3 weeks to obtain a dynamic picture of COVID knowledge and related behaviours and household outcomes (see Supplementary material). The data have been used to evaluate the impact of non-pharmaceutical interventions implemented by government policy.^6^

## What has it found?

**Figure 2:**
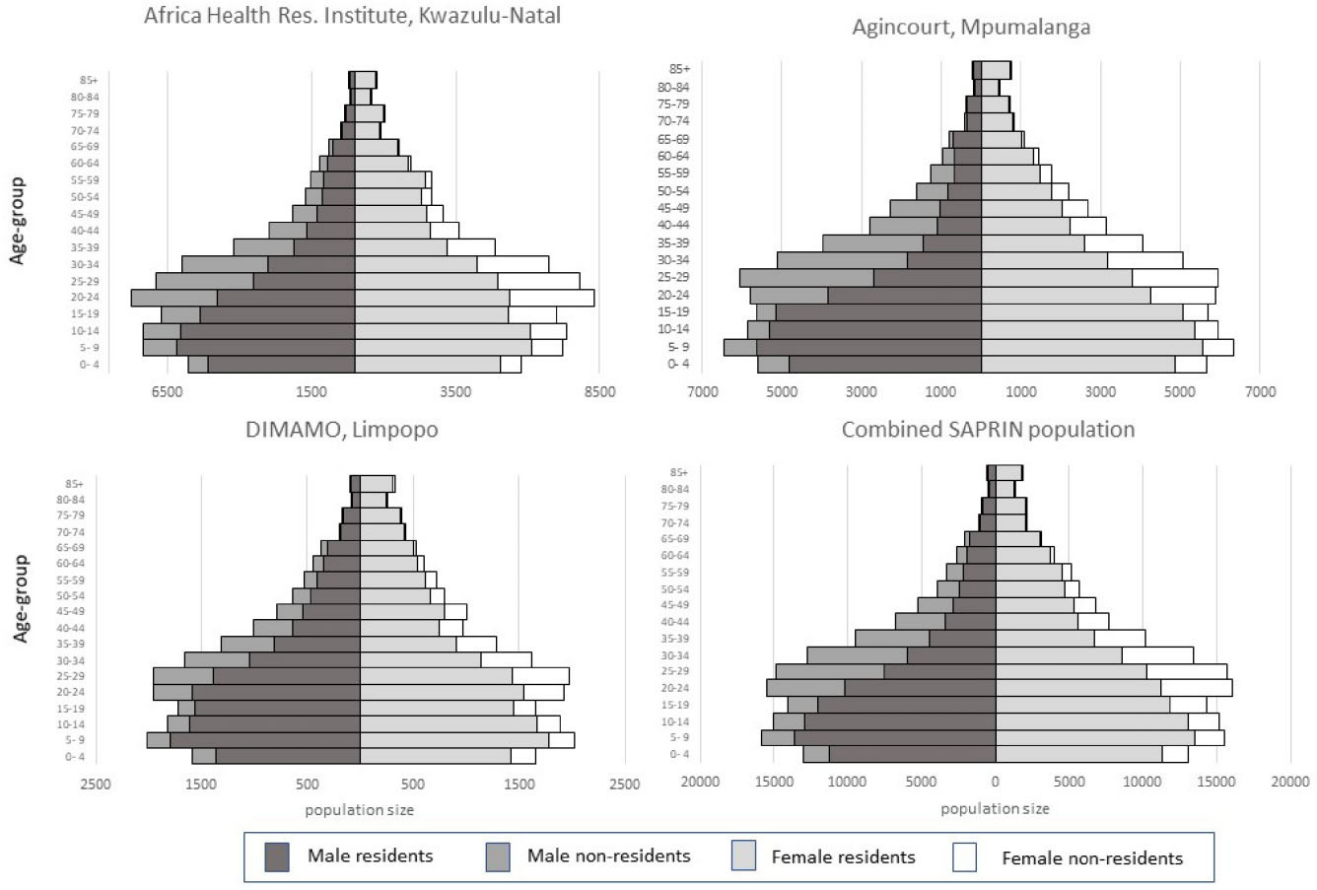
Age-sex-residency population pyramids on 1 July 2017 for the three SAPRIN nodes separately and the combined population

**Figure 3:**
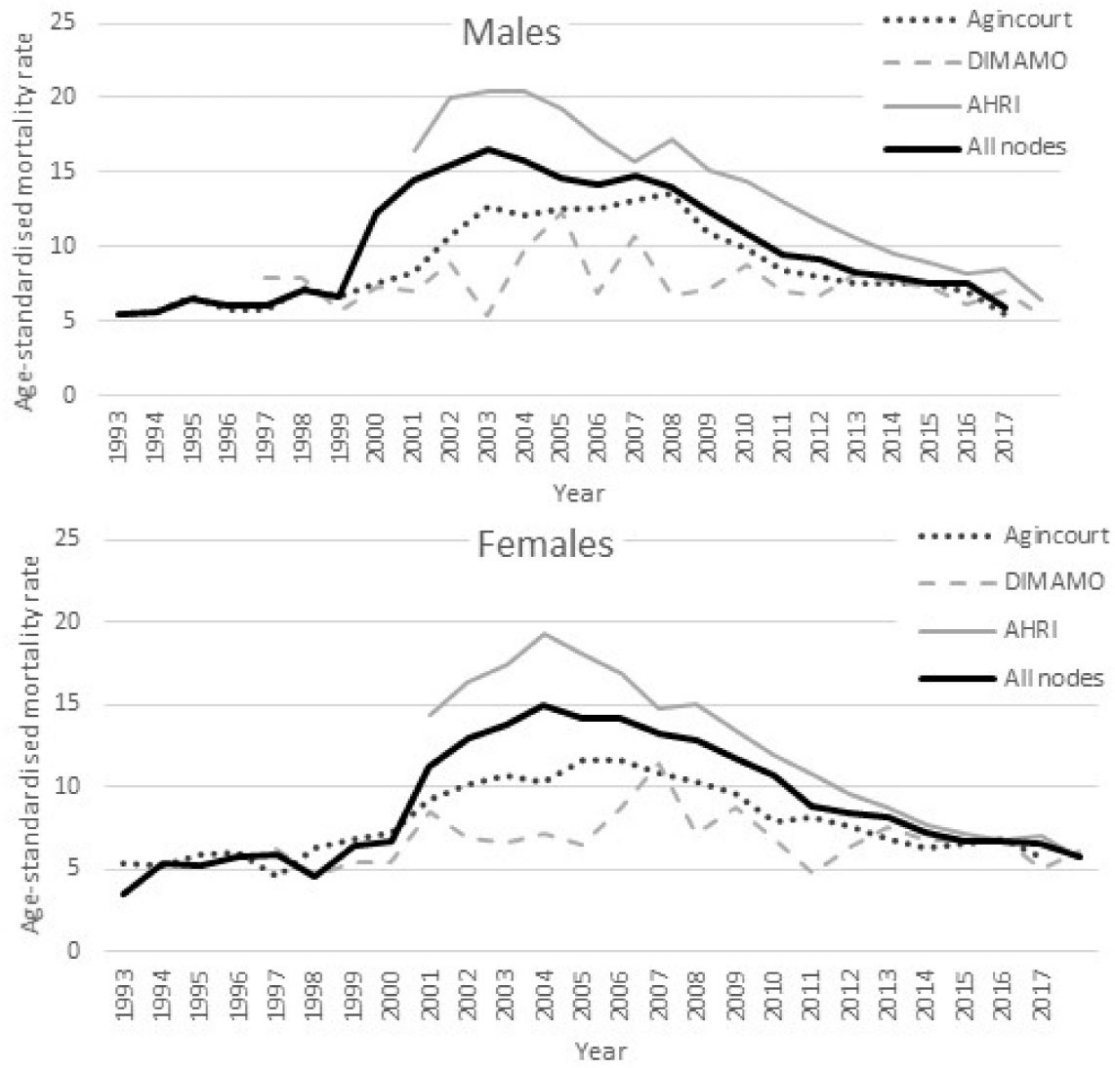
Age-standardized mortality rate per 1000 individuals for males and females for the three SAPRIN nodes separately and combined

**Figure 4:**
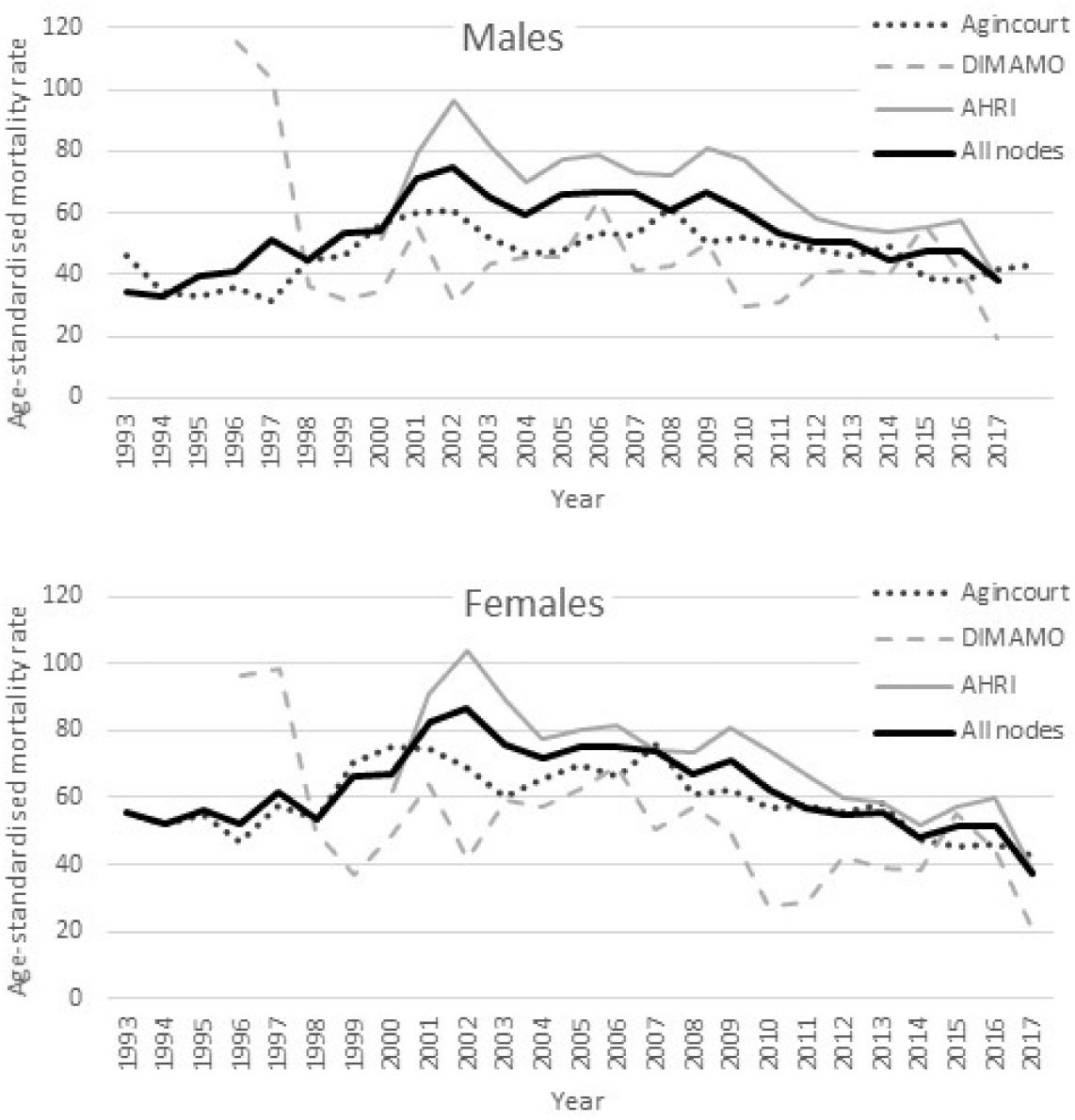
Age-standardized in-migration rate per 1000 individuals for males and females for the three SAPRIN nodes separately and combined

**Figure 5:**
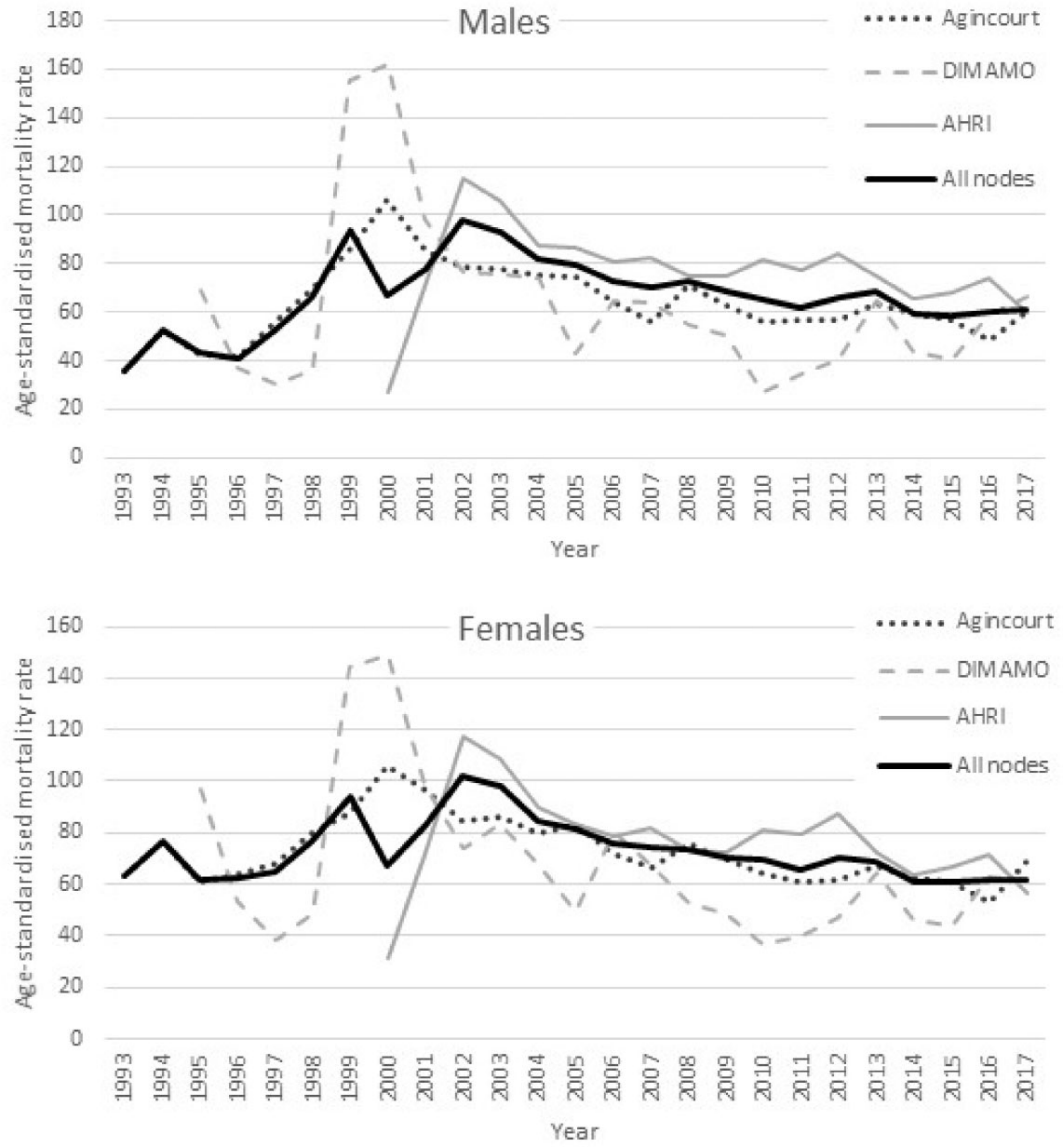
Age-standardized out-migration rate per 1000 individuals for males and females for the three SAPRIN nodes separately and combined

Figures 2–5 were computed from the basic residency dataset at the SAPRIN Data Repository.^7^ The plots below were computed from the basic residency dataset [https://doi.org/10.23667/SAPRIN.SISEBD2020], downloaded from the SAPRIN Data Repository on 15 June 2020.

### Population dynamics

Age-sex pyramids were constructed from harmonised HDSS data on the same day (1 July 2017) at the three SAPRIN nodes. Their similarity, despite coming from different provinces which exhibit heterogeneity of living environments, employment opportunities and culture, indicates that the nodal data can be combined. A comparison of the age/sex structure in the Agincourt population with the national census 2011 shows a good fit. Common features include the large sub-population of youth aged 20–25 years and a higher female sex ratio in older ages.^8^

The population pyramids include resident and non-resident household members, which reveals the extent of temporary, largely labour migration. With a median age of 20–24 years, there is a similar distribution of temporary migrants in the three nodes, balanced by sex. The temporary migration levels are especially high in the KwaZulu-Natal node. The least prominent temporary migrant age groups are in the teenage years, due to school attendance, and after the age of 60 when migrants retire, are eligible for a government pension and return to their rural homes. The location of all three nodes in former ‘Bantustans’ highlights the extreme lack of work opportunities in rural areas due to distance from labour markets. DIMAMO is closer to an urban centre, Polokwane, which explains why the proportion of temporary migrants of working ages is lower.

### Mortality trends

Despite age standardization there are major shifts in mortality profiles over time. The series start at different points aligned with different starts of the HDSS operations: 1993 in Agincourt, 1997 in DIMAMO and 2000 in AHRI. The AIDS-TB epidemic affected all the populations, with an especially high impact and prevalence in KwaZulu-Natal.^9^ The HDSS findings highlight the decline in mortality associated with improved public health interventions introduced at different times by the respective provincial departments of health. These are supported by research contributed to by the SAPRIN nodes.^10–13^

### Migration trends

Migration is defined here as moving into the dwelling of a household in a SAPRIN node from outside the node or moving out from a dwelling within the node to outside the node. As above, the migration trends begin in different years, as data became available. For all nodes there were high peaks of migration around the turn of the millennium, from 1999–2002, followed by a slow decline in migration rates for men and women over a 15-year period, with the lowest levels being current. Due to high levels of circularity, the periods with high migration rates have higher both in- and out-migration rates.^14^

### Embedded research

The platform provides the opportunity for a range of observational and interventional study designs. Table 3 shows the current research data themes at each node, arising from their respective research portfolios.

**Table 3:** Nodal research data themes

SAMRC/Wits Agincourt Rural Public Health and Health Transitions Research Unit	DIMAMO Population Health Research Centre	Africa Health Research Institute
Research, trials and evaluation platform, including health, population and social transitions Respiratory virus transmission studies, including SARS-CoV-2, influenza, RSV Adolescent health and development, including HIV prevention, risk factors for chronic disease, anxiety and depression Adult and older adult health and well-being, including NCD and infectious chronic diseases, multimorbidity, cognition and dementias Social and environmental determinants of health, including migration, urbanization and health; and natural resources and food security	Non-communicable diseases Mental health Strengthening the response to dementia Intervention to optimize adolescent BMI pre-conception, to address the double burden of malnutrition Impact of COVID-19 on household food security, nutritional status and socioeconomic status COVID–19 risk communication and community engagement Population studies	HIV epidemiology HIV prevention and treatment trials TB epidemiology Chronic disease multimorbidity COVID-19 immune mechanisms Social epidemiology Population studies

SAMRC, South African Medical Research Council; DIMAMO, an amalgam of the initial two letters of the three sub-districts covered by the field site; RSV, respiratory syncytial virus; NCD, non-communicable disease; BM, body mass index; TB, tuberculosis.

The work shows increased cardiometabolic disease risk across the life course, with early stunting in children (one-third of 1-year-olds) coupled with adolescent overweight and obesity (20–25% in older girls).^15^ The MRC/Wits-Agincourt Unit’s HAALSI (Health and Aging in Africa: Longitudinal Studies of an INDEPTH community) cohort was designed to examine the biological, social and economic conditions that shape health in the ageing population.^16^ High blood pressure and obesity in middle-aged women are at unprecedented levels, fostered by changes in lifestyle, diet and occupation.^17–20^ The effect of dementia and cognitive impairment on HIV status knowledge is high. Taken together, the associations with literacy and cognitive function are even more important than educational attainment in predicting HIV status knowledge.^21^ A comparison of HIV prevalence in older people, comparing Agincourt and AHRI, showed rates above 15% for men and 10% for women through to age 70 in both populations.^22^

South Africa (SA) has the largest HIV prevention and antiretroviral treatment programme (ART) in the world with almost 8 million people living with HIV, of whom 5 million (62%) are on treatment. In 2017, the country spent R30 billion on HIV-related health services. SAPRIN nodes, especially the AHRI node, contributed key insights ranging from quantifying improved life expectancy since the introduction of ART, to showing how new HIV infections declined by 43% in 2012–17.^23^ This demonstrates the impact of government prevention and treatment programmes and provides invaluable information for planning.

The Africa-Wits-INDEPTH Partnership for Genomic Studies (AWI-Gen), within DIMAMO Population Health Research Centre (PHRC) and the MRC/Wits-Agincourt Unit, are collaborative Centres of the H_3_Africa Consortium. AWI-Gen is aimed at studying the genomic and environmental risk factors for cardiometabolic disease in Africans. Furthermore, the DIMAMO PHRC has partnered with the University of Cape Town to conduct a study on strengthening response to dementia in developing countries (STRIDE). The study is aimed at improving dementia care, treatment and support systems.

Internal migration linking rural and urban areas is highly prevalent in rural South Africa, is central to the national economy and impacts on everyday life. Both benefits and costs need to be understood to make sure migrants are safe and healthy. Health risks can be infectious, such as HIV or COVID-19, or non-communicable, such as hypertension.^24–26^ When migrants are away from home, they can get sick or injured at the destination and come home for health care and attention, which can be a burden on the rural home and health system.^27^

When the COVID-19 pandemic reached South Africa in March 2020, SAPRIN illustrated the benefits of having a population platform in place to react quickly to the scientific and social challenges posed by this global threat. By the beginning of April 2020 SAPRIN had an approved COVID-19 surveillance protocol in place, and by mid-April 2020 a long-term observational cohort was established that covered COVID-19 knowledge and behaviour and the economic and health impacts, including mental health, of the national lockdown.

## What are the main strengths and weaknesses?

The important strengths of HDSS data come from their longitudinal structure and complete coverage, and from being representative of under-resourced and vulnerable populations in which reliable health, population and socioeconomic data are scarce. With dates of birth, death and in- and out-migration captured following the initial baseline census, the population dynamics are tracked on a day-by-day basis. Time episodes are recorded of each person’s residence in a study village dwelling place, as well as membership of a social unit, namely a household. Non-residential household membership is recorded, which means that the HDSS platform can distinguish between permanent and temporary migration. As an analytical method, continuous time event history analysis can readily be conducted with any of the population dynamic variables, births, deaths and in- and out-migration (for both permanent and temporary migration).^28^

Weaknesses also arise from the intensity and high detail of data collection. The generalizability of findings to a national population is not a step that can be taken simply, because the HDSS does not have a study design to ensure this. The characteristics of the sub-population need to be understood in the national context. Nevertheless, some studies show that small-area population data can represent national mortality patterns quite well.^29^

Another limitation is that data collection for lengthy periods and participation in multiple trials can produce a ‘Hawthorne Effect’, whereby study communities that are rigorously observed can be changed by the effect of their participation, and a control group may not be absent from intervention effects obtained in another trial.^30^^,^^31^

The burden for participants of repeated rounds of data collection is coupled with expectations of local and direct benefits to individuals and communities linked to the research. Communities are by necessity partners in the work and are routinely involved in events where research results are fed back and implications discussed.^32–34^

## Can I get hold of the data? Where can I find out more?

The SAPRIN longitudinal population datasets are publicly available and can be accessed from [http://saprindata.samrc.ac.za]. Requests for other data or information can be made to the authors via e-mail (mark.collinson@mrc.ac.za).

The core protocol for SAPRIN is conducted in accordance with the Declaration of Helsinki^35^ and the principles of Good Clinical Practice as laid down in the ICH Harmonised Tripartite Guideline for Good Clinical Practice.^36^ The protocol was approved (protocol: EC010-3/2021) by the Human Research Ethics Committee (HREC) of the South African Medical Research Council (SAMRC). In addition, each node obtains ethical review and oversight from their local HREC for studies embedded in the research platform.

## Supplementary Data

Supplementary data are available at *IJE* online.

## Funding

SAPRIN is funded by the Department of Science and Innovation (DSI) as part of the South African Research Infrastructure Road Map (SARIR) and is hosted by the South African Medical Research Council (SAMRC).

## Supplementary Material

dyab261_Supplementary_Data
