# Supplementary material for: Cohort Profile: South African Population Research Infrastructure Network (SAPRIN)
Source: Int J Epidemiol. 2021 Dec 30;51(4):e206–16. doi: 10.1093/ije/dyab261 (PMC9365637; doi:10.1093/ije/dyab261)
Supplement: dyab261_Supplementary_Data [file dyab261_supplementary_data.docx]

| **Supplementary file — Variables collected at SAPRIN HDSS nodes** | | |
| --- | --- | --- |
| **Variable** | **Description** | |
| 1. Base Entities | The core entities on which longitudinal data will be collected. Each entity has a dated start and end event | |
| - 1. Location | A place to which geo-coordinates can be assigned | |
| - - 1. Type | 1. Dwelling. A place of residence within the boundaries of the demographic surveillance area 2. Clinic. A primary health care facility, without overnight facilities 3. Health Centre. A primary health care facility that is larger than a clinic with some overnight care facilities 4. Hospital 5. Primary School. Teaching up Grade 7 6. Secondary School. Teaching from Grade 8 to 12 7. Creche. A pre-school childcare facility 8. Police station 9. Tribal authority 10. Social service office 11. Home affairs office | |
| - - 1. Area | Village or other higher level spatial grouping of locations | |
| - - 1. Coordinate | Latitude and longitude on the WGS84 coordinate system | |
| - 1. Household | A household resident at a location within the boundary of the surveillance area. A household is defined as a social group of one or more individual members. Resident household members share the same dwelling as the household, non-resident household members are individuals who do not share the same dwelling as the household, but who eat from the same pot when they are present in the household’s dwelling. | |
| - - 1. Identifier | A unique household identifier which the household retains irrespective of its current residence | |
| - 1. Individual | Household members, where a **resident** is a member of a household who normally lives (i.e. intends to sleep the majority of the time) at the same dwelling as the household; and a **non-resident** is a member of a household who does not normally live at the same dwelling as the household but is nevertheless considered a member of the household. | |
| - - 1. Identifier | A globally unique identifier used internally to uniquely identify the individual | |
| - - 1. External identifier | An identifier used on data collection instruments to uniquely identify the individual, but is removed together with other personally identifiable information when data is made available for analysis | |
| - - 1. Surname | Family or last name | |
| - - 1. Alternative surname | An alternative surname (formerly) used by the individual, e.g. maiden name | |
| - - 1. First name 1 | The first given name of the individual | |
| - - 1. First name 2 | The second/alternative given name of the individual | |
| - - 1. Civilian ID number | The 13-digit South African civilian identity number | |
| - - 1. Citizenship | The country of which the individual is a citizen | |
| - - 1. Nationality | The country of origin of the individual (the country of which the person is/was a citizen by birth) | |
| - - 1. Sex | Male (1), female(2) or unknown(0) | |
| - - 1. Birth | The Birth event of this individual or the DeliveryEvent resulting in the birth of this individual if the individual enters surveillance through birth | |
| - - 1. EndEvent | The death event of this individual or the Observation at which this individual was last observed | |
| - - 1. MotherId | The unique identifier of the mother of the individual, if the mother is enumerated in the surveillance | |
| - - 1. FatherId | The unique identifier of the father of the individual, if the father is enumerated in the surveillance | |
| - - 1. MotherSurname | The surname of the mother of the individual | |
| - - 1. MotherFirstName | The first name/s of the mother | |
| - - 1. FatherSurname | The surname of the father of the individual | |
| - - 1. FatherFirstName | The first name/s of the father | |
| 1. Events | Demographic events that delineate episodes of observation during longitudinal surveillance | |
| - 1. Migration | A change in location of usual residence | |
| - - 1. Date | Date of migration | |
| - - 1. Type | 1. Internal (change of location within surveillance area) 2. External (change of location into/out of the surveillance area) | |
| - - 1. Direction | 1. In (migration into this location) 2. Out (migration out of this location) | |
| - - 1. Origin | Origin of the migration (location) | |
| - - 1. Destination | Destination of the migration (location) | |
| - - 1. Unit | Individual or household | |
| - 1. Birth | The birth event of an individual | |
| - - 1. Date | Date of birth | |
| - - 1. Birth weight | Birth weight as obtained from the Road to Health card in grams | |
| - - 1. BirthCertificate | Whether the birth certificate was observed | |
| - - 1. DateRegistered | The date on which the birth was registered, or null if the birth hasn’t been registered yet | |
| - 1. PregnancyOutcome | The end event of a pregnancy | |
| - - 1. Date | Date of birth | |
| - - 1. Type | 1. Spontaneous abortion 2. Assisted abortion 3. Caesarean 4. Assisted 5. Normal vaginal   Types i-ii is only applicable if the pregnancy end prior to the 28^th^ week of pregnancy, types iii-v is applicable only on or after the 28^th^ week of pregnancy | |
| - - 1. Place | 1. In a health care facility 2. Outside a health care facility, but not at home 3. At home | |
| - - 1. BirthAttendant | 1. Doctor 2. Midwife 3. Traditional birth attendant 4. Lay person | |
| - - 1. LiveBirths | The number of babies born alive resulting from this delivery | |
| - - 1. Stillborn | The number of still born babies resulting from this delivery | |
| - 1. Death | The death of an individual | |
| 1. Date | The date of the death | |
| 1. Place | 1. In a health care facility 2. Outside a health care facility, but not at home (road, etc) 3. At home | |
| 1. DeathCertificate | Observed | |
| 1. Verbal autopsy | WHO standard verbal autopsy questionnaire items. SAPRIN will adopt the worldwide standardised World Health Organisation (WHO) Verbal Autopsy questionnaires to determine the cause of deaths reported in the surveillance population. The latest version of the WHO Verbal Autopsy Questionnaire is 2016, edited and cognitively tested to facilitate the use of publicly available analytical software for assigning the cause of death. | |
| - 1. Enumeration | The initial event at enumeration of an entity. Can only be used during the base census of the demographic surveillance, or when a new area is added to the surveillance | |
| - - 1. Date | Date of enumeration | |
| - 1. Membership | A change in household membership | |
| - - 1. Date | The date of the change | |
| - - 1. Type | 1. Start 2. End 3. Household dissolution | |
| - 1. Household Headship | A change in the head of the household | |
| - - 1. Date | The date of the change | |
| - - 1. Type | 1. Start 2. End | |
| - 1. UnionEvent | A change in the union (conjugal relationship) between two individuals | |
| - - 1. Date | The date of the event | |
| - - 1. Type | 1. Start 2. Marriage 3. Separation 4. Divorce 5. Partner died | |
| - 1. Observation | A surveillance visit at a location | |
| - - 1. Date | Visit date | |
| - - 1. Location | Location where the visit took place | |
| - - 1. DataCollector | The person responsible for data collection during the visit | |
| - - 1. Respondent | The primary respondent at the visit | |
| 1. Episodes | Used to record associations longitudinally between base entities. Episodes are always started and ended through events. | |
| - 1. Residence | An episode during which an individual is resident (sleeping most of the time there) at a particular location that falls within the surveillance area. An individual can only be resident at one location at a time, i.e. residency episodes cannot overlap | |
| - - 1. Individual | The individual identifier | |
| - - 1. Location | The location identifier | |
| - - 1. StartEvent | The event that started the episode, can only be Enumeration, Birth, Migration (Direction: In) | |
| - - 1. EndEvent | The event that terminated the episode, can only be Death, Migration (Direction: Out) and Observation (in which the implication is that the individual is last known to be resident) | |
| - 1. HouseholdResidence | An episode during which a household is resident at a particular location that falls within the surveillance area. A household can only be resident at one location at a time, i.e. residency episodes cannot overlap | |
| - - 1. Household | The household identifier | |
| - - 1. Location | The location identifier | |
| - - 1. External identifier | The identifier associated with the household during this residence | |
| - - 1. StartEvent | The event that started the episode, can only be Enumeration, Household formation, Migration (Direction: In) | |
| - - 1. EndEvent | The event that terminated the episode, can only be Household dissolution, Migration (Direction: Out) and Observation (in which the implication is that the household is last known to be resident) | |
| - 1. Membership | An episode during which an individual is a member of a household. An individual must be a member of at least one household to be under surveillance. An individual can be a member of more than one household at a time (membership episodes may overlap). In the case of multiple memberships, the designated household of an individual will be the household the individual is co-resident with, ranked according to the closeness of the members relationship to the household head (self, spouse, child, grandchild, parent, sibling, other relationship) | |
| - - 1. Individual | The individual identifier | |
| - - 1. Household | The household identifier | |
| - - 1. StartEvent | The event that started the episode, can only be Enumeration, Birth or Membership start | |
| - - 1. EndEvent | The event that ended the episode, can only be Death, Membership end or Observation (implying that the membership is current) | |
| - 1. Household Head Relationship | Household head relationships are linked to a household membership and record the relationship between the individual and the head of the associated household. If the head of household change, all current household members start a new household head relationship episode | |
| - - 1. Membership | The household membership episode associated with this household head relationship | |
| - - 1. Relationship | The relationship between the individual member (to whom the membership belongs) and the current household head of the household   1. Self (the individual who is the subject of the membership is the household head) 2. Spouse (incl partner in stable relationship) 3. Child (incl adopted/foster child) 4. Son/daughter-in-law (incl individuals in stable relationship with any child of the household head) 5. Grandchild 6. Parent 7. Parent-in-law (incl parent of partner in stable relationship) 8. Grandparent 9. Other relative 10. Domestic worker or tenant 11. Unrelated household member | |
| - - 1. StartEvent | The event that started the episode. Can only be Enumeration, Birth, Membership start, or Household Headship start | |
| - - 1. EndEvent | The event that ended the episode. Can only be Death, Membership end, Household Headship end or Observation | |
| - 1. Union | The episode during which two persons are in an informal or formal conjugal relationship. For a conjugal relationship to exist the following factors should be considered:   1. Shelter. Do the partners live under the same roof? 2. Sexual and personal conduct. Do the partners have sexual relations; do they maintain an attitude of fidelity to each other; do they eat their meals together? 3. Services. Do they share household responsibilities? 4. Social. Do they participate together in social activities; does their society recognise them as a couple? 5. Support. Do they support each other financially? 6. Children. Do they have children together? | |
| - - 1. Individual1 | The individual identifier of one of the parties to the union. By convention this will be the female in a heterosexual union. | |
| - - 1. Individual2 | The individual identifier of the second party to the union. | |
| - - 1. StartEvent | The start of the union. Can only be Enumeration or Union start | |
| - - 1. MarriageDate | The date on which the union has been formalised as a marriage | |
| - - 1. EndEvent | The end of the union. Can only be Union – partner died, Union – separation (if there is no marriage date), Union – divorce (if there is a marriage date), or Observation if it a current union | |
| - 1. Pregnancy | The period of being pregnant. Also used to record maternity histories retrospectively | |
| - - 1. Woman | The individual identifier of the woman who experienced the pregnancy | |
| - - 1. ANCVisits | The number of antenatal care visits during this pregnancy | |
| - - 1. Duration | Duration in weeks of the pregnancy | |
| - - 1. Outcome | A delivery event or Observation if the pregnancy is still current at the time of observation | |
| - 1. Social support | The period during which an individual receives a government social support grant | |
| - - 1. Grantholder | The individual identifier of the person holding the grant | |
| - - 1. Beneficiary | The individual identifier of the intended beneficiary of the grant. This may be the same as the identifier of the grant holder in the case where the holder is the beneficiary, e.g. old age pension, or different as in the case of care dependency grants, where the grant holder receives the grant on behalf of someone else, e.g. a child | |
| - - 1. Type | The type of grant   1. Old age 2. Disability 3. War veterans 4. Care dependency 5. Foster child 6. Child support 7. Grant-in-aid | |
| - - 1. StartDate | The start date of the social support | |
| - - 1. EndDate | The end date of the social support | |
| 1. StatusObservation | Information collected at a particular observation, valid only at the time of the observation. State may be imputed between consecutive status observations but is not known to be valid. This is in contrast with episode where the assertion is that the state represented by the episode is valid for the duration of the episode. | |
| - 1. Individual StatusObservation | A set of data elements collected about an individual either during a face-to-face visit or telephonic interview with the individual or from a proxy informant | |
| - - 1. Individual | The unique individual identifier of the subject of the status observation | |
| - - 1. Observation | The observation at which the status observation was made | |
| - - 1. ResidentStatus | Physical presence in the dwelling, recorded as the number of months since the previous observation visit | |
| - - 1. MotherStatus | 1. Same household 2. Same area (village/isigodi) 3. Elsewhere in surveillance area 4. In the immediate surroundings outside surveillance area 5. Elsewhere 6. Died 7. Unknown status | |
| - - 1. FatherStatus | 1. Same household 2. Same area (village/isigodi) 3. Elsewhere in surveillance area 4. In the immediate surroundings outside surveillance area 5. Elsewhere 6. Died 7. Unknown status | |
| - - 1. HighestSchoolLevelCompleted | Grade 1 – 12 | |
| - - 1. HighestNonSchoolEducation | 1. Undergraduate degree 2. Postgraduate degree 3. ABET 1-4 4. NQF 1-4 | |
| - - 1. CurrentEducation | If the individual is currently attending an educational institution, at what level:   1. Creche 2. Pre-school 3. Grade 1-12 4. ABET 1-4 5. NQF 1-4 6. Undergraduate degree 7. Post-graduate degree 8. Not attending | |
| - - 1. Currently employed | 1. Yes 2. Part-time 3. No | |
| - - 1. Not employed | Type/reason for unemployment   1. Caring for others/household duties 2. Looking for work 3. Student or in training 4. Unable to work due to illness/disability 5. Other reason | |
| - - 1. EmploymentSector | 1. Agriculture/Fishing/Forestry 2. Mining 3. Manufacturing 4. Electricity and water 5. Construction 6. Wholesale/retail 7. Restaurant/Hotels/Sport/Tourism 8. Transport and communication 9. Finance 10. Educational services 11. Health services 12. Legal services 13. Research 14. Domestic services 15. Armed forces 16. Informal sector, e.g. street vendor | |
| - - 1. EmploymentType | 1. Works as employee 2. Work for themselves 3. Do odd jobs/piece jobs | |
| - - 1. Employer | 1. Central government 2. Provincial administration 3. Local / regional authority 4. Public corporation 5. Private sector employer 6. Non-profit institution 7. Self-employment 8. Another household member | |
| - - 1. FinancialStatus | Self-reported financial status   1. Very Comfortable 2. Comfortable 3. Just Getting By 4. Poor 5. Extremely Poor | |
| - - 1. MaritalStatus | 1. Never married 2. Married 3. Polygamous Marriage 4. Divorced/Separated 5. Widowed | |
| - - 1. PartnershipStatus | 1. Marital Partnership 2. Regular Partnership 3. Casual Partnership(s) 4. No Partnership | |
| - - 1. HealthStatus | Self-reported health status   1. Excellent, Very Good or Good 2. Fair 3. Poor | |
| - - 1. Tuberculosis | 1. Ever treated - Yes/No 2. Treatment started in last 12 months – Yes/No 3. Currently on TB treatment – Yes/No | |
| - - 1. HIV | 1. Ever received a test result for HIV - Yes/No 2. Ever had a positive HIV result – Yes/No | |
| - - 1. If HIV+ | 1. When first HIV+ result (>1yr, <1yr ago) 2. When last HIV- result (>1yr, <1yr ago) 3. When first started ART (Never, <1yr, >1yr) 4. Currently on ART (Yes/No) | |
| - - 1. If HIV- | 1. When last HIV- result (>1yr, <1yr ago) | |
| - - 1. HIVResult | HIV serostatus from dried blood spot | |
| - - 1. Hypertension | 1. Ever treated - Yes/No 2. Treatment started in last 12mos 3. Currently on treatment | |
| - - 1. Diabetes | 1. Ever treated - Yes/No 2. Treatment started in last 12mos - Yes/No 3. Currently on treatment - Yes/No | |
| - - 1. Health care utilisation | 1. Admitted to hospital past month - Yes/No 2. Visited a clinic past month – Yes/No 3. Visited private doctor past month – Yes/No 4. Used pharmacy/chemist past month – Yes/No 5. Visited traditional healer past month – Yes/No | |
| - - 1. Vaccination history (child <=6yr) | For each vaccination record: date received and source of information (Road to Health Card or recall)   1. At birth – BCG & Polio 0 2. At 6w – Polio1, DTab+IPV+HiB1, HepB1, Rota1, PCV1 3. At 10w – DtaP+IPV+Hib2, HepB2 4. At 14w – DtaP+IPV+Hib3, HepB3, Rota2, PCV2 5. At 9mos – Measles1, PCV3 6. At 18mos – DtaP+IPV+Hib4, Measles2 7. At 6yr - DT | |
|  |  | |
| - 1. Household Status Observation | Set of data elements collected from a household informant, during a face to face or telephonic interview | |
| - - 1. Water source | The most commonly used (during last year) source of drinking water   1. Piped – to stand/house 2. Piped – Public tap/kiosk 3. Borehole/well 4. Rainwater 5. Flowing river/stream 6. Dam/standing water 7. Protected spring 8. Water carrier or tanker | |
| - - 1. Toilet | What kind of toilet does the household use   1. Flush 2. Ventilated improved pit 3. Other pit 4. Bucket 5. Chemical | |
| - - 1. Electricity supply | Is the household connected to the electricity grid?   1. Yes 2. No | |
| - - 1. Cooking fuel | What is the main fuel used for cooking?   1. Wood 2. Gas (LPG) 3. Coal 4. Electricity | |
| - - 1. Dwelling construction | What is the construction materials of the walls?   1. Brick 2. Cement 3. Other modern building material 4. Stabilised mud 5. Traditional mud 6. Wood 7. Other informal structures   What is the construction material of the floor?   1. Tiles 2. Cement 3. Modern carpet 4. Wood 5. Other modern material 6. Dirt 7. Mat 8. Other traditional   How many bedrooms does your household occupy at this dwelling? | |
| - - 1. Assets | Does the household have any of the following items in good working order? | |
|  | 1. Telephone 2. Cellphone 3. Primus Cooker, Sikeni 4. Electric hot plate 5. Electric stove with oven 6. Gas Cooker 7. Fridge/Freezer 8. Electric kettle 9. Television set 10. Video cassette recorder/DVD 11. Radio/Stereo 12. Sewing machine 13. Block maker | 1. Car or Bakkie 2. Motorcycle/Scooter 3. Bicycle 4. Kombi/Lorry/Tractor 5. Bed 6. Table and chairs 7. Sofa/Sofa set 8. Kitchen sink 9. Car battery for Electricty 10. Wheelbarrow 11. Hoe,Spade or Garden Fork 12. Bed Nets 13. Cattle 14. Other Livestock (chickens etc) |
| - - 1. Crime | Has any resident member of the household been a victim of any of these crimes in the past 12 months?   1. None 2. Theft 3. Assault 4. Murder 5. Other crime | |
| - - 1. Financial situation | How would the household classify its financial situation these days?   1. Very comfortable 2. Comfortable 3. Just getting by 4. Poor 5. Extremely poor | |
| - - 1. Food security | In the last 12 months did you or any other member of your household ever cut the size of your meals or skip meals because there was not enough money for food?   1. Yes – how often (Almost every month, some months but not all, only once or twice) 2. No | |
| 1. **Covid-19** | **Set of data elements on Covid-19 Surveillance collected from a household informant or individual during a face to face or telephonic interview** | |
| 1. Household actions as result of Covid-19 | \| 1. Avoided crowded areas 2. Avoided socializing 3. Avoided church 4. Avoided going shopping (markets, stores) 5. Avoided going to restaurants/bars/pubs 6. Avoided going to doctor/health centre/clinic 7. Avoided going to school/university 8. Avoided taking taxis 9. Avoided going outside 10. Avoided going to work \| 1. Avoided travelling long distances 2. Tried to avoid physical contact with people 3. Avoided family members 4. Avoided friends 5. Washed hands more often 6. Avoided touching own face 7. Used face masks 8. Used gloves 9. Used hand sanitizer 10. Other (specify) 11. Nothing \| \| --- \| --- \| | |
| 1. Knowledge of Covid-19 pandemic | 1. Less than I should know 2. A little, but not enough 3. Enough 4. A little more than most people 5. I am up to date on the latest research | |
| 1. Source of trusted Covid-19 information | 1. Newspapers/new websites 2. Radio 3. TV 4. Government websites 5. Social media (Facebook, Twitter, YouTube) 6. Forwarded email, WhatsApp 7. Other websites 8. Friends, family, colleagues 9. Posters, billboards 10. Health workers, clinic materials 11. Traditional healers 12. School 13. Church 14. Community leaders 15. Other (specify) | |
| 1. Any visitors to household yesterday | 1. Yes 2. No 3. Don’t know | |
| 1. Any household member taking daily medication for long-term health condition | 1. Yes 2. No 3. Don’t know | |
| 1. Every in household taking daily medication for long-term health condition had access last 7 days | 1. Yes 2. No 3. Don’t know | |
| 1. Main reason why someone in household not able to access daily medication last 7 days | 1. Afraid of get medication because of COVID-19; 2. Lockdown restricted me from going out; 3. Unable to go out for other reasons; 4. Medicine out of stock; 5. Unable to afford medication; 6. Other (specify) | |
| 1. Anyone moved into household address last 4 months due to Covid-19 | 1. Yes 2. No | |
| 1. Anyone left household address since March 2020 due to Covid-19 | 1. Yes 2. No | |
| 1. Any household member unable to access healthcare in past 7 days | 1. Yes 2. No 3. Don’t know | |
| 1. Main reason unable to access healthcare | 1. Afraid to go out due to COVID-19 2. Lockdown restricted me from going out 3. Unable to go out for other reason 4. Unable to afford 5. Clinic closed 6. Other (specify) | |
| 1. Household able to get food necessities past 7 days | 1. Yes 2. No 3. Don’t know | |
| 1. Household current financial situation compared to before Covid-19 lockdown | 1. Much worse off; 2. A little worse off; 3. About the same; 4. A little better off; 5. Much better off | |
| 1. In past 30 days has any sources of household income decreased | 1. Employment income – lost job; 2. Employment income – unpaid leave; 3. Business income; 4. Government grants; 5. Money from friends or family; 6. Other (specify) 7. Household had no income before; 8. No income source has decreased | |
| 1. Household received any Covid-19 support past 30 days | 1. Yes 2. No 3. Don’t know | |
| 1. How children accessed education past 7 days | 1. Aa 2. In person at school 3. Through a school-based internet program 4. Through TV/radio programs at home 5. From household members at home 6. They received no education 7. It has been school holidays | |
| 1. Household experienced difficulties providing childcare past 7 days | 1. Yes, a lot 2. Yes, a little 3. No | |
| 1. Things in community changed for better or worse since March 2020 | 1. Got better; 2. stayed the same; 3. got worse | |
| 1. Things in community changed for better or worse since March 2020 | 1. Got better; 2. stayed the same; 3. got worse | |
| 1. Living arrangement changed because of the COVID-19 pandemic | 1. Yes 2. No | |
| 1. Changed how | 1. I moved to my current address temporarily; 2. Another person (e.g. sibling, adult, child, parent) has moved into my address; 3. Another person I lived with has moved elsewhere 4. Other (specify) | |
| 1. Compared to before Covid-19 how violence against women changed | 1. Increased; 2. Stayed the same; 3. Decreased; 4. Don’t know | |
| 1. Compared to before Covid-19 how violence against men changed | 1. Increased; 2. Stayed the same; 3. Decreased; 4. Don’t know | |
| 1. Compared to before Covid-19 how violence against children changed | 1. Increased; 2. Stayed the same; 3. Decreased; 4. Don’t know | |
| 1. Compared to before Covid-19 how child neglect changed | 1. Increased; 2. Stayed the same; 3. Decreased; 4. Don’t know | |
| 1. Compared to before Covid-19 marital problems changed | 1. Increased; 2. Stayed the same; 3. Decreased; 4. Don’t know | |
| 1. People with Covid-19 deserve to be punished | 1. Strongly disagree; 2. Disagree; 3. Agree; 4. Strongly agree | |
| 1. People with Covid-19 are irresponsible | 1. Strongly disagree; 2. Disagree; 3. Agree; 4. Strongly agree | |
| 1. People with Covid-19 bring shame on their families | 1. Strongly disagree; 2. Disagree; 3. Agree; 4. Strongly agree | |
| 1. People with Covid-19 are dirty | 1. Strongly disagree; 2. Disagree; 3. Agree; 4. Strongly agree | |
| 1. People with Covid-19 are cursed | 1. Strongly disagree; 2. Disagree; 3. Agree; 4. Strongly agree | |
| 1. Afraid to visit someone recovered from Covid-19 | 1. Strongly disagree; 2. Disagree; 3. Agree; 4. Strongly agree | |
| 1. If I get Covid friend or family would be angry at me | 1. Very unlikely; 2. Unlikely; 3. Likely; 4. Very likely | |
| 1. If I get Covid friend or family would be blame me | 1. Very unlikely; 2. Unlikely; 3. Likely; 4. Very likely | |
| 1. If I get Covid friend or family would say it’s my fault | 1. Very unlikely; 2. Unlikely; 3. Likely; 4. Very likely | |
| 1. If I get Covid friend or family employer would fire me | 1. Very unlikely; 2. Unlikely; 3. Likely; 4. Very likely | |
| 1. If I get Covid workmates will discriminate me | 1. Very unlikely; 2. Unlikely; 3. Likely; 4. Very likely | |
| 1. If I get Covid workmate will blame me | 1. Very unlikely; 2. Unlikely; 3. Likely; 4. Very likely | |
| 1. If I get Covid it will be hard to tell other people | 1. Very unlikely; 2. Unlikely; 3. Likely; 4. Very likely | |
| 1. If I get Covid I will feel ashamed | 1. Very unlikely; 2. Unlikely; 3. Likely; 4. Very likely | |
| 1. Ever been tested for COVID-19? | 1. Yes; 2. No; 3. Don’t know | |
| 1. previously diagnosed with COVID-19? | 1. Yes; 2. No; 3. Don’t know | |
| 1. When diagnosed with COVID-19? | 1. Yes; 2. No; 3. Don’t know | |
| 1. Hospitalized for COVID-19? | 1. Yes; 2. No; 3. Don’t know | |
| 1. Vaccinated against Covid-19 | 1. Yes; 2. No; 3. Don’t know | |
| 1. When received first dose? | 1. Date | |
| 1. Which vaccine received first dose? | 1. Jansen/J&J 2. Pfizer/BioNTech 3. Moderna 4. Astra-Zeneca/Oxford/ChadOx 5. Don’t know 6. Other (specify) | |
| 1. Where received first dose? | 1. Local clinic/hospital names; 2. private practitioner, 3. chemist, 4. mobile vaccine facility (place) | |
| 1. Received a second dose? | 1. Yes; 2. No; | |
| 1. When received 2nd dose? | 1. Date | |
| 1. Which vaccine 2nd first dose? | 1. Jansen/J&J 2. Pfizer/BioNTech 3. Moderna 4. Astra-Zeneca/Oxford/ChadOx 5. Don’t know 6. Other (specify) | |
| 1. Where received 2nd dose? | 1. Local clinic/hospital names; 2. private practitioner, 3. chemist, 4. mobile vaccine facility (place) | |
| 1. Reasons choosing to vaccinate against COVID-19? | 1. To protect others in my family; 2. To protect my community from COVID-19; 3. To protect myself from getting sick with COVID-19; 4. I have a chronic health condition; 5. If my doctor, nurse or health worker recommends it; 6. It would be the best way to avoid getting seriously ill from COVID-19; 7. It would allow me to feel safe around other people; 8. Life won’t go back to normal until most people are vaccinated; 9. Other (soecify) 10. None | |
| 1. Reasons choosing not to vaccinate against COVID-19? | 1. I am allergic to vaccines; 2. I don’t like needles; 3. I don’t get vaccines in general; 4. People in my community do not get vaccines in general; 5. I’m not concerned about getting ill from the coronavirus; 6. I would be concerned about getting infected with the coronavirus from the vaccine; 7. I am concerned about side effects from the vaccine; 8. I don’t think that the vaccine will work; 9. The coronavirus outbreak is not as serious as some people say it is; 10. It might be expensive; 11. It will be too late because we will have already been infected; 12. I expect vaccination site will require long/expensive travel; 13. I expect vaccination site will be open inconvenient hours/require long wait times; 14. Other (specify) 15. None | |
| 1. Have any of the following symptoms currently? | 1. Fever ≥ 38°C; 2. Cough; 3. Chills; 4. Sore throat; 5. Shortness of breath / breathing difficulties; 6. Nausea / Vomiting; 7. Diarrhoea; 8. Myalgia / Body pains; 9. General weakness; 10. Irritability / Confusion; 11. Loss of taste (ageusia); 12. Loss of sense of smell (anosmia); 13. Other (specify) 14. None | |
| 1. If <2 years old, additional symptoms? | 1. Lethargy; 2. Poor feeding; 3. None | |
| 1. earliest date of symptoms onset | 1. Date | |
| 1. Close physical contact with known COVID-19 case? | 1. Yes; 2. No; 3. Don't Know | |
| 1. Contact setting | 1. Healthcare setting; 2. Household setting; 3. Workplace; 4. Public transport setting; 5. Other (specify) | |
| 1. Left home past 7 days? | 1. Yes; 2. No; 3. Don't Know | |
| 1. Left village/suburb past 7 days | 1. Yes; 2. No; 3. Don't Know | |
| 1. Travelled to local town past 7 days? | 1. Yes; 2. No; 3. Don't Know | |
| 1. Travelled beyond subdistrict past 7 days? | 1. Yes; 2. No; 3. Don't Know | |
| 1. Why left home? | 1. To get food/medication; 2. To go to work; 3. To visit friends or family; 4. To get exercise; 5. To provide care to a vulnerable person; 6. Other (specify) | |
| 1. Having little interest or pleasure in doing things past 14 days? | 1. Not at all; 2. Several days; 3. More than half the days; 4. Nearly every day | |
| 1. Feeling down, depressed or hopeless past 14 days? | 1. Not at all; 2. Several days; 3. More than half the days; 4. Nearly every day | |
| 1. Feeling nervous, anxious or on edge past 14 days? | 1. Not at all; 2. Several days; 3. More than half the days; 4. Nearly every day | |
| 1. Not able to stop or control worrying past 14 days? | 1. Not at all; 2. Several days; 3. More than half the days; 4. Nearly every day | |
| 1. Likelihood getting Covid? | 1. Certain; 2. Very likely; 3. Somewhat likely; 4. Not very likely; 5. No chance at all; 6. I’ve already had Covid-19; 7. Don’t know | |
| 1. Know someone diagnosed as having COVID-19? | 1. Yes; 2. No; 3. Don't Know | |
| 1. concerned about exposure to COVID-19? | 1. Yes; 2. No; 3. Don't Know | |
| 1. Cannot trust government tell truth about Covid-19 | 1. Strongly agree; 2. Agree; 3. Neither agree nor disagree; 4. Disagree; 5. Strongly disagree | |
| 1. trust public health measures government is taking to combat Covid-19 | 1. Strongly agree; 2. Agree; 3. Neither agree nor disagree; 4. Disagree; 5. Strongly disagree | |
| 1. Government not giving enough clear information about Covid-19 | 1. Strongly agree; 2. Agree; 3. Neither agree nor disagree; 4. Disagree; 5. Strongly disagree | |
| 1. coronavirus info withheld from public | 1. Strongly agree; 2. Agree; 3. Neither agree nor disagree; 4. Disagree; 5. Strongly disagree | |
